# Supplementary material for: Modeling glioblastoma heterogeneity as a dynamic network of cell states
Source: Mol Syst Biol. 2021 Sep 16;17(9):e10105. doi: 10.15252/msb.202010105 (PMC8444284; doi:10.15252/msb.202010105)
Supplement: Supplementary file 6 — Source Data for Figure 5 [file MSB-17-e10105-s004.zip › Figure5A_sourcedata/GSEA_3071/hallmarks_stateV.GseaPreranked.1623742546329/HALLMARK_CHOLESTEROL_HOMEOSTASIS.html]

Details for gene set HALLMARK\_CHOLESTEROL\_HOMEOSTASIS[GSEA]

|  || Dataset | state43071 |
| Phenotype | NoPhenotypeAvailable |
| Upregulated in class | na\_neg |
| GeneSet | HALLMARK\_CHOLESTEROL\_HOMEOSTASIS |
| Enrichment Score (ES) | -0.481206 |
| Normalized Enrichment Score (NES) | -2.0917368 |
| Nominal p-value | 0.0 |
| FDR q-value | 5.808261E-4 |
| FWER p-Value | 0.005 |
Table: GSEA Results Summary

  

Fig 1: Enrichment plot: HALLMARK\_CHOLESTEROL\_HOMEOSTASIS      
 Profile of the Running ES Score & Positions of GeneSet Members on the Rank Ordered List

  

| PROBE | GENE SYMBOL | GENE\_TITLE | RANK IN GENE LIST | RANK METRIC SCORE | RUNNING ES | CORE ENRICHMENT || 1 | CLU |  |  | 464 | 0.900 | -0.0473 | No |
| 2 | FABP5 |  |  | 589 | 0.806 | -0.0343 | No |
| 3 | CD9 |  |  | 738 | 0.728 | -0.0293 | No |
| 4 | TMEM97 |  |  | 963 | 0.636 | -0.0430 | No |
| 5 | GLDC |  |  | 1245 | 0.547 | -0.0716 | No |
| 6 | TM7SF2 |  |  | 1273 | 0.539 | -0.0523 | No |
| 7 | GUSB |  |  | 2060 | 0.379 | -0.1844 | No |
| 8 | FADS2 |  |  | 2155 | 0.367 | -0.1856 | No |
| 9 | PDK3 |  |  | 2707 | 0.298 | -0.2767 | No |
| 10 | CPEB2 |  |  | 2744 | 0.295 | -0.2702 | No |
| 11 | NSDHL |  |  | 3434 | -0.288 | -0.3880 | No |
| 12 | IDI1 |  |  | 3680 | -0.328 | -0.4197 | No |
| 13 | NFIL3 |  |  | 3700 | -0.331 | -0.4083 | No |
| 14 | SQLE |  |  | 4082 | -0.405 | -0.4623 | Yes |
| 15 | LSS |  |  | 4157 | -0.426 | -0.4570 | Yes |
| 16 | HMGCR |  |  | 4221 | -0.446 | -0.4487 | Yes |
| 17 | STARD4 |  |  | 4393 | -0.499 | -0.4586 | Yes |
| 18 | EBP |  |  | 4441 | -0.517 | -0.4440 | Yes |
| 19 | ATF5 |  |  | 4467 | -0.525 | -0.4250 | Yes |
| 20 | MVD |  |  | 4604 | -0.591 | -0.4240 | Yes |
| 21 | ANTXR2 |  |  | 4608 | -0.593 | -0.3976 | Yes |
| 22 | ANXA5 |  |  | 4616 | -0.601 | -0.3717 | Yes |
| 23 | HMGCS1 |  |  | 4646 | -0.616 | -0.3492 | Yes |
| 24 | PNRC1 |  |  | 4813 | -0.726 | -0.3478 | Yes |
| 25 | ALCAM |  |  | 4988 | -0.911 | -0.3395 | Yes |
| 26 | ACTG1 |  |  | 5017 | -0.961 | -0.3012 | Yes |
| 27 | CYP51A1 |  |  | 5037 | -1.013 | -0.2589 | Yes |
| 28 | LGALS3 |  |  | 5039 | -1.017 | -0.2129 | Yes |
| 29 | TRIB3 |  |  | 5148 | -1.313 | -0.1738 | Yes |
| 30 | GPX8 |  |  | 5171 | -1.418 | -0.1136 | Yes |
| 31 | PLAUR |  |  | 5185 | -1.479 | -0.0490 | Yes |
| 32 | S100A11 |  |  | 5196 | -1.536 | 0.0188 | Yes |
Table: GSEA details [plain text format]

  

Fig 2: HALLMARK\_CHOLESTEROL\_HOMEOSTASIS: Random ES distribution      
 Gene set null distribution of ES for **HALLMARK\_CHOLESTEROL\_HOMEOSTASIS**
